# Supplementary figures and images for: Metabolic Characteristics of Gut Microbiota and Insomnia: Evidence from a Mendelian Randomization Analysis
Source: Nutrients. 2024 Sep 2;16(17):2943. doi: 10.3390/nu16172943 (PMC11397146; doi:10.3390/nu16172943)

A

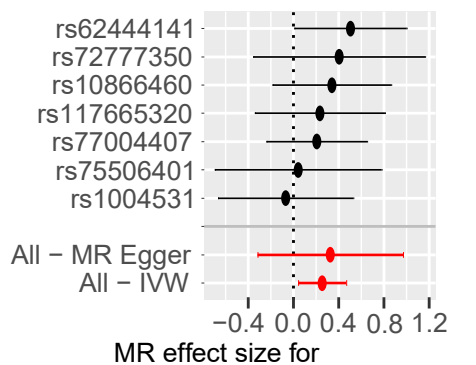

B

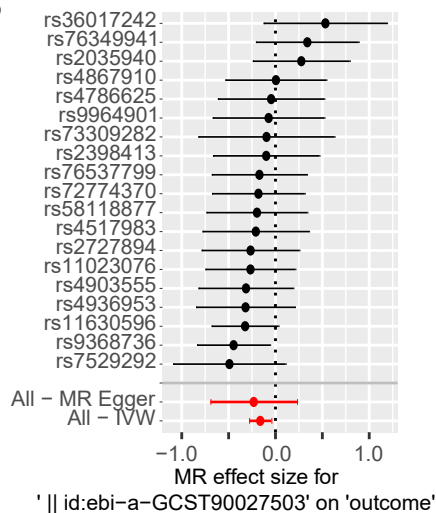

C

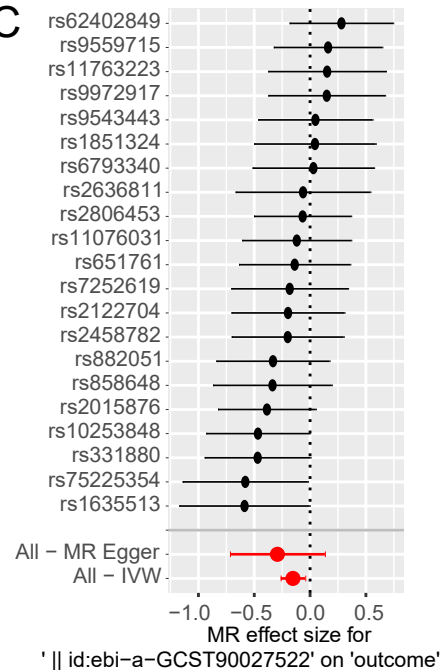

D

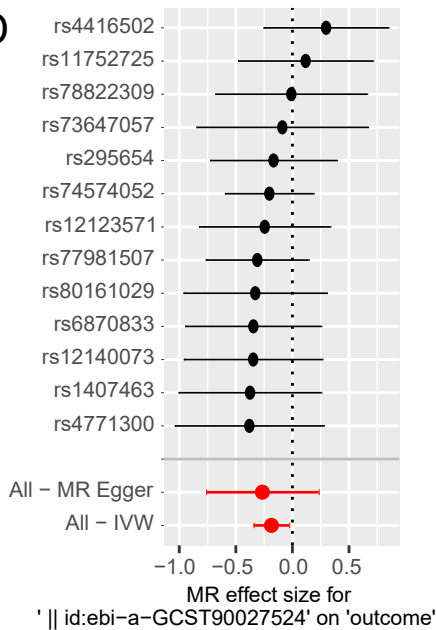

E

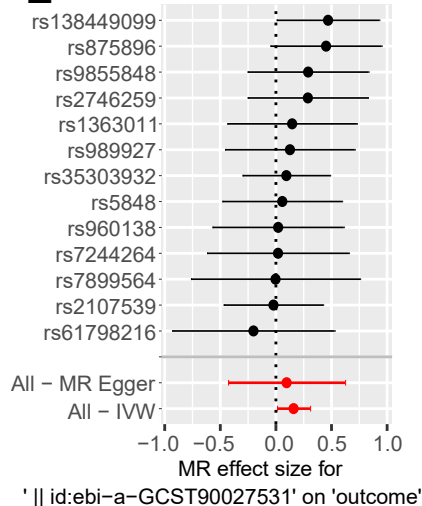

F

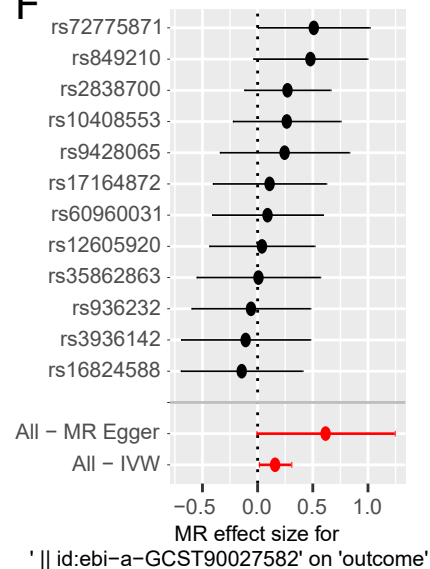

G

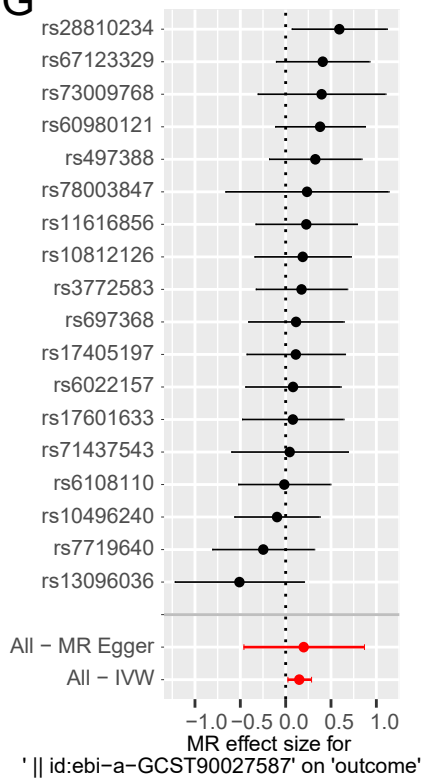

H

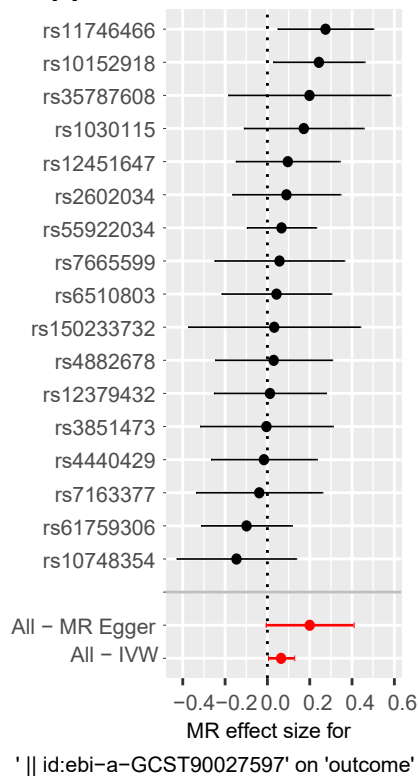

I

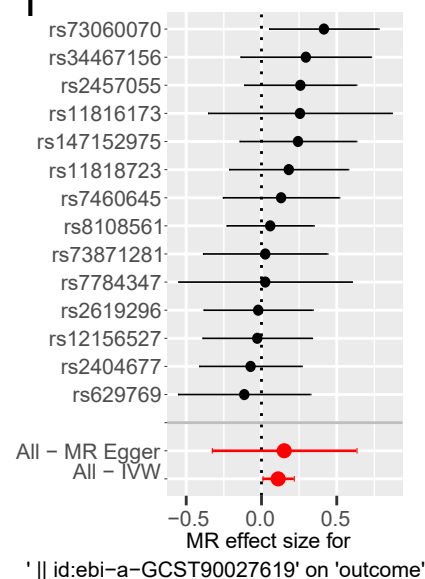

Supplement: Supplementary file 1 [file nutrients-16-02943-s001.zip › Figure S1 The causal effect of single SNP in the direction with significant MR results.pdf]

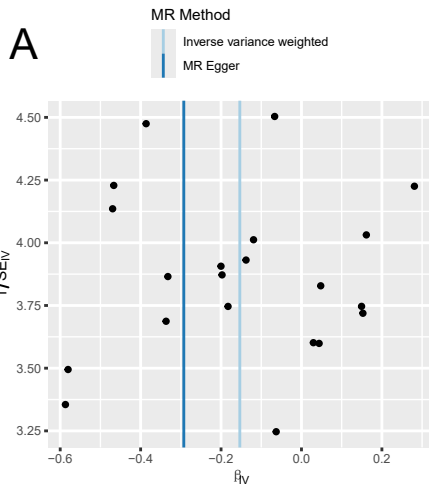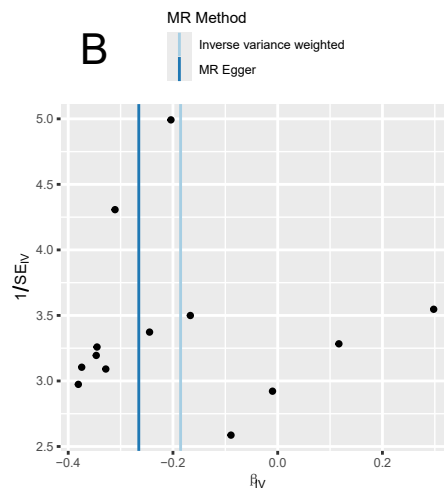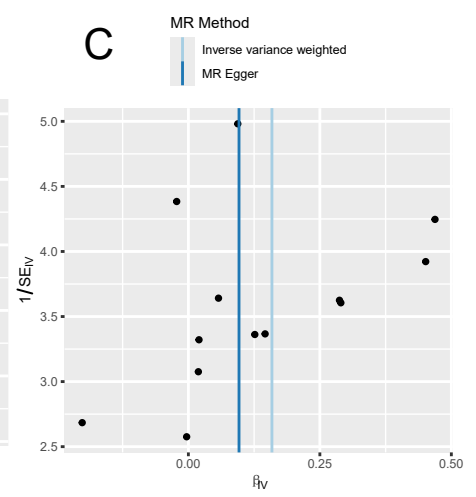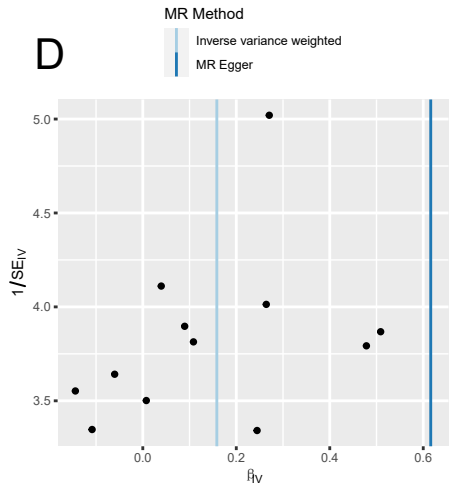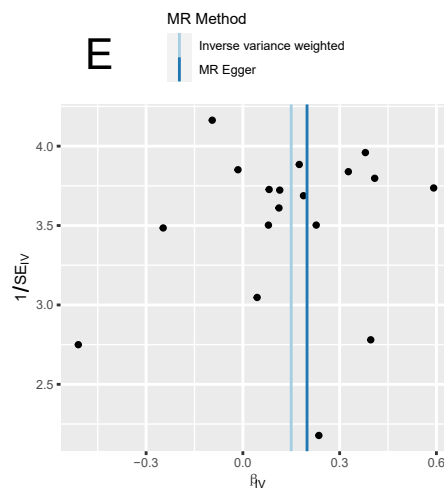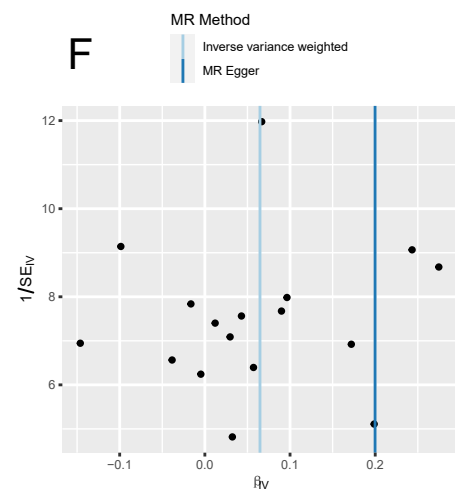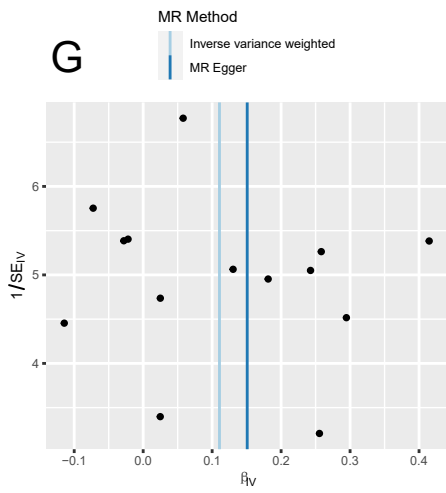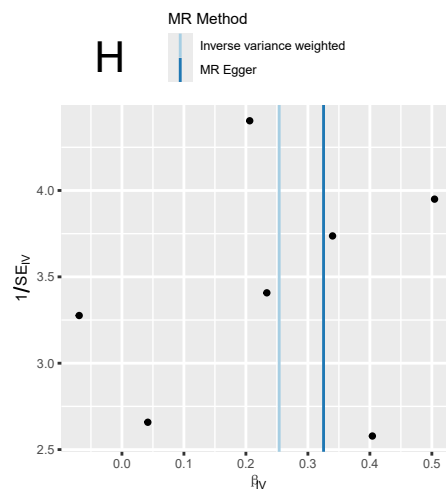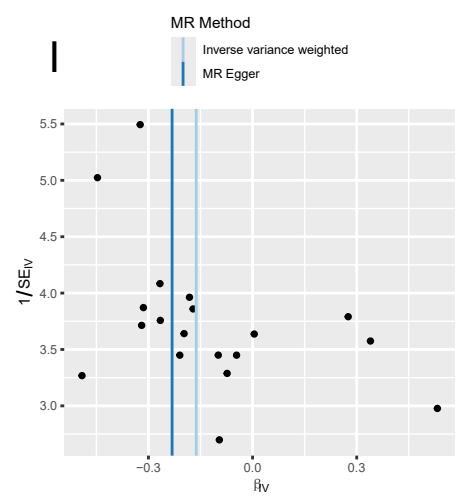

Supplement: Supplementary file 1 [file nutrients-16-02943-s001.zip › Figure S2 Funnle plots for the directions with significant MR analysis.pdf]
